# Time trends in the prevalence and epidemiological characteristics of neural tube defects in Liaoning Province, China, 2006-2015: A population-based study

**DOI:** 10.18632/oncotarget.15060

**Published:** 2017-02-03

**Authors:** Tie-Ning Zhang, Ting-Ting Gong, Yan-Ling Chen, Qi-Jun Wu, Yuan Zhang, Cheng-Zhi Jiang, Jing Li, Li-Li Li, Chen Zhou, Yan-Hong Huang

**Affiliations:** ^1^ Department of Pediatrics, Shengjing Hospital of China Medical University, Shenyang, China; ^2^ Department of Obstetrics and Gynecology, Shengjing Hospital of China Medical University, Shenyang, China; ^3^ Liaoning Women and Children’s Health Hospital, Shenyang, China; ^4^ Department of Clinical Epidemiology, Shengjing Hospital of China Medical University, Shenyang, China; ^5^ Department of Emergency, Shengjing Hospital of China Medical University, Shenyang, China; ^6^ School of Environmental and Chemical Engineering, Shenyang Ligong University, Shenyang, China; ^7^ Department of Science and Education, Shenyang Women and Children Health Care Centre, Shenyang, China; ^8^ Department of Children's Health Prevention, Shenyang Women and Children Health Care Centre, Shenyang, China; ^9^ Department of Information Statistics, Shenyang Women and Children Health Care Centre, Shenyang, China

**Keywords:** epidemiology, neural tube defects, perinatal, prevalence, time trend

## Abstract

To evaluate the time trends in the prevalence of neural tube defects and all their subtypes as well as to identify the epidemiological characteristics of these malformations documented in the Liaoning Province of northeast China from 2006 to 2015. This was a population-based observational study using data from 3,248,954 live births as well as from 6217 cases of neural tube defects, 1,600 cases of anencephaly, 2,029 cases of spina bifida, 404 cases of encephalocele, and 3,008 cases of congenital hydrocephalus from 14 cities in Liaoning Province from 2006 to 2015. All analyses were conducted using SPSS software. During the observational period, the prevalence of neural tube defects, anencephaly, spina bifida, encephalocele, and congenital hydrocephalus was 19.1, 4.9, 6.2, 1.2, and 9.3 per 10,000 live births, respectively. Significantly decreasing trends were observed in the prevalence of all these malformations except for encephalocele. Notably, relatively higher prevalence rates were found in isolated compared with non-isolated malformations, with significant differences in selected characteristics (e.g., prognosis status, gestational age, and birth weight) between isolated and non-isolated cases of these malformations. The prevalence of neural tube defects showed a downward trend in Liaoning Province from 2006 to 2015. However, more attention should be focused on non-isolated cases in the future because of the severe clinical manifestations. Future prevention efforts should be strengthened to reduce the risk of these malformations, especially the non-isolated subtype, in areas with high prevalence.

## INTRODUCTION

Neural tube defects (NTDs), are a group of serious birth defects caused by abnormal development of the neural tube during embryonic life that produces injuries of the brain and spinal cord [1]. The majority of NTDs cases are diagnosed prenatally, and termination of pregnancy for fetal anomaly has become the most common outcome [2–4]. Because of the severe outcomes of this malformation, many studies have been conducted to investigate the mechanisms, and findings suggest that the development of this disease is a multistep process strictly controlled by genes and modulated by a host of environmental factors [5]. Recently, conclusive evidence from clinical trials has led to recommendations for adequate periconceptional folic acid intake to reduce the occurrence of an NTD-affected pregnancy [6].

A recent systematic review [7] suggested great variability in reported NTD prevalence estimates globally (range, 0.3–199.4 per 10,000 births). Of note, almost 80% of the studies reported prevalence estimates above 6.0 per 10,000 births. When stratified by subtypes of NTDs, spina bifida contributed the highest percentage, followed by anencephaly and then encephalocele. However, the data used to describe the time trends in the prevalence of NTDs in the majority of these studies were collected one or two decades ago. A similar phenomenon was also observed in China. For example, the prevalence of NTDs was 199.4 per 10,000 live births in Luliang Prefecture, a city in the Shanxi Province of China, from 2004 to 2005 [8]. By contrast, from 2003 to 2009, the prevalence was 0.3 per 10,000 live births in Beijing [9]. Although a recent study conducted by Liu and colleagues [10] showed that the prevalence of NTDs presented a continuous decreasing trend and was 31.5 per 10,000 live births in 2014 in the Shanxi Province of China. However, data reflecting the status of NTDs in China during the last decade have been quite limited for other areas. Such information would be critical to understand whether similar decreasing trends hold for other regions. The prevalence of NTDs in the past decade as well as whether similar trends can be observed in other cities remains unknown. Notably, no recent study has described the time trend in the prevalence of NTDs and their subtypes (anencephaly, spina bifida, encephalocele, and congenital hydrocephalus) or stratified these malformations by whether they are accompanied by additional major defects (i.e., isolated versus non-isolated cases of malformations).

Liaoning Province, which encompasses an area of 145,900 square kilometers and has a population of almost 42 million, has contributed greatly to the development of China in the past decades. Nevertheless, no formal assessment of this population has been made. Therefore, to evaluate the time trends in the prevalence of NTD subtypes during the recent decade as well as to identify the epidemiological characteristics of these malformations, we conducted a population-based study using the most recent database for Liaoning Province for the 10-year period from 2006 to 2015.

## RESULTS

**Figure 1:**
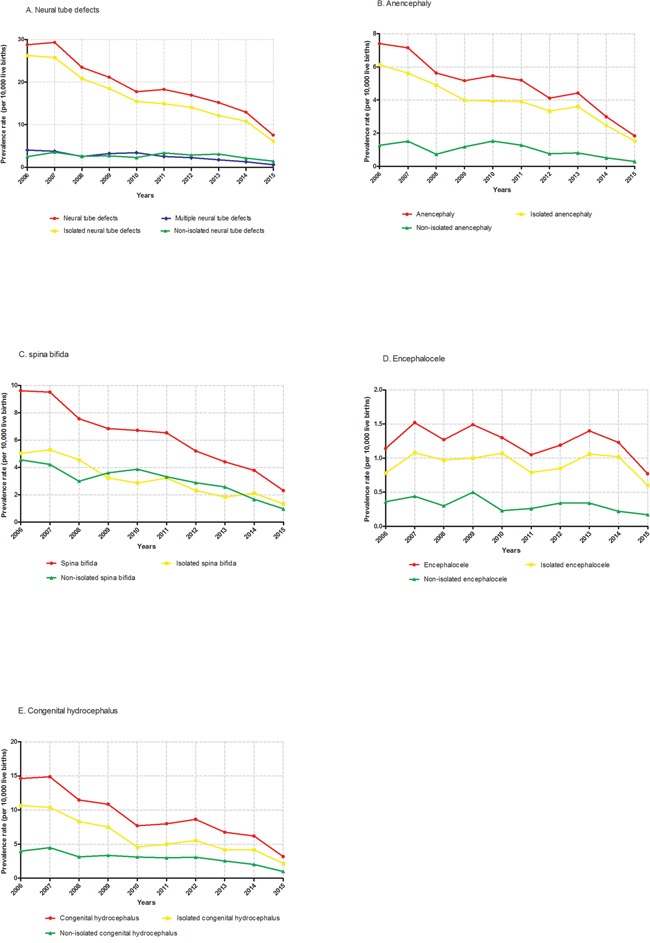
Time trends in the prevalence of NTDs in Liaoning Province from 2006 to 2015 **A**. neural tube defects; **B**. anencephaly; **C**. spina bifida; **D**. encephalocele; **E**. congenital hydrocephalus.

Table 1 presents the results of the time-trend analysis for the prevalence of NTDs in Liaoning Province from 2006 to 2015. A total of 6217 NTDs cases were detected among 3,248,954 live births (prevalence rate, 19.1 per 10,000 live births). Compared with congenital hydrocephalus (9.3 per 10,000 live births), a relatively lower prevalence was observed for anencephaly (4.9 per 10,000 live births), spina bifida (6.2 per 10,000 live births), and encephalocele (1.2 per 10,000 live births). Except for spina bifida, significantly higher prevalence was detected in isolated compared with non-isolated malformations (Table 1). Figure 1 visually depicts the time trends in the prevalence of NTDs in Liaoning Province during the observational period. Except for encephalocele and non-isolated NTDs, a significant decrease was observed in the prevalence of all NTDs (Table 1), with the prevalence rate for NTDs, anencephaly, spina bifida, and congenital hydrocephalus significantly decreased annually by 10.68%, 10.15%, 11.57%, and 11.84%, respectively. Notably, the extent of the decrease observed in the prevalence of congenital hydrocephalus included both the greatest and least annual change (13.24% versus 8.79%). The greatest contribution to the decrease in NTDs was congenital hydrocephalus, which accounted for 33.3% of the overall decrease (Table 2). By contrast, encephalocele had the lowest contribution rate. Additionally, except for encephalocele, all isolated malformations had relatively higher contribution rates than non-isolated ones.

**Table 1:** The prevalence and time trends of neural tube defects (per 10,000 births)

	Year										Overall	AverageChange (%)	*P*value	95% CI
	2006	2007	2008	2009	2010	2011	2012	2013	2014	2015				
**NTDs**	28.8	29.3	23.4	21.1	17.7	18.3	16.9	15.2	12.9	7.5	19.1	−10.68	<0.001	−13.32, −7.97
Multiple NTDs	4.0	3.7	2.5	3.2	3.4	2.5	2.2	1.7	1.3	0.6	2.5	−12.19	0.001	−17.49, −6.55
Isolated	26.3	25.7	20.8	18.5	15.5	14.9	14.0	12.1	10.8	6.1	16.5	−11.93	<0.001	−14.13, −9.66
Non-isolated	2.5	3.5	2.6	2.7	2.3	3.4	2.9	3.1	2.1	1.4	2.7	−3.15	0.25	−8.79, 2.84
**Anencephaly**	7.4	7.1	5.6	5.2	5.5	5.2	4.1	4.4	3.0	1.8	4.9	−10.15	<0.001	−13.60, −6.56
Isolated	6.1	5.6	4.9	4.0	3.9	3.9	3.3	3.6	2.5	1.5	3.9	−10.33	<0.001	−13.38, −7.17
Non-isolated	1.3	1.5	0.7	1.2	1.5	1.3	0.8	0.8	0.5	0.3	1.0	−9.52	0.04	−17.49, −0.77
**Spina bifida**	9.6	9.5	7.6	6.8	6.7	6.5	5.2	4.4	3.8	2.3	6.2	−11.57	<0.001	−14.19, −8.88
Isolated	5.1	5.3	4.6	3.2	2.9	3.2	2.3	1.8	2.1	1.3	3.2	−13.06	<0.001	−16.02, −10.00
Non-isolated	4.6	4.2	3.0	3.6	3.9	3.3	2.9	2.6	1.7	1.0	3.1	−9.88	0.003	−14.73, −4.75
**Encephalocele**	1.1	1.5	1.3	1.5	1.3	1.1	1.2	1.4	1.2	0.8	1.2	−2.57	0.21	−6.74, 1.80
Isolated	0.8	1.1	1.0	1.0	1.1	0.8	0.8	1.1	1.0	0.6	0.9	−1.19	0.55	−5.65, 3.47
Non-isolated	0.4	0.4	0.3	0.5	0.2	0.3	0.3	0.3	0.2	0.2	0.3	−6.39	0.06	−12.85, 0.55
**CH**	14.6	14.9	11.5	10.9	7.7	8.0	8.6	6.8	6.2	3.2	9.3	−11.84	<0.001	−15.03, −8.52
Isolated	10.7	10.4	8.3	7.5	4.6	5.0	5.6	4.2	4.2	2.2	6.3	−13.24	<0.001	−16.57, −9.77
Non-isolated	4.0	4.5	3.1	3.4	3.1	3.0	3.1	2.6	2.0	1.0	3.0	−8.79	0.002	−13.10, −4.26

CI, confidence interval; CH, congenital hydrocephalus.

**Table 2:** The relative contributions of decreasing trend of neural tube defects prevalence

Birth Defects	Decreasing trend	
	β	Contribution rate (%)
**NTDs**		
Anencephaly	−0.11	28.2
Spina bifida	−0.12	30.8
Encephalocele	−0.03	7.7
Congenital hydrocephalus	−0.13	33.3
**NTDs**		
Isolated	−0.13	81.3
Non-isolated	−0.03	18.7
**Anencephaly**		
Isolated	−0.11	52.4
Non-isolated	−0.10	47.6
**Spina bifida**		
Isolated	−0.14	58.3
Non-isolated	−0.10	41.7
**Encephalocele**		
Isolated	−0.01	12.5
Non-isolated	−0.07	87.5
**Congenital hydrocephalus**		
Isolated	−0.14	60.9
Non-isolated	−0.09	39.1

The characteristics of mothers and infants with NTDs are shown in Table 3. For time to diagnosis, the majority of these cases were diagnosed during pregnancy. For prognosis status, inducing labor was the major outcome, which accounted for 76.4%, 85.2%, 68.5%, 76.2%, and 78.9% in NTDs, anencephaly, spina bifida, encephalocele, and congenital hydrocephalus, respectively. Additionally, anencephaly had the lowest proportion of live births, whereas spina bifida had the highest. The majority of the mothers was Han, aged 20 to 30 years, and had an income level over 2400 yuan/person and an education level of middle school. The majority of the infants with malformations were singletons with a gestational age of less than 28 weeks and a birth weight less than 2,500 grams. Besides, when comparing the epidemiological characteristics for NTDs and its subtypes by multivariable analysis, significant differences were existed in selected characteristics when treating encephalocele as reference group (Table 3). For example, significant differences were observed in all three subtypes regarding time of diagnosis, number of live birth, gestational age, and birth weight. Additionally, three (prognosis status, maternal age, and sex) and one characteristic (educational level) showed significance in two and one subtype of NTDs, respectively.

**Table 3:** Characteristics for neural tube defects and their subtypes of infants and their mother *

Characteristics	NTDs	Anencephaly	Spina bifida	Encephalocele	Congenital hydrocephalus
**No. of cases**	6217	1600	2029	404	3008
**Time of diagnosis (%)**					
During pregnancy	5651 (90.9)	1580 (98.8)	1663 (82.0)	368 (91.1)	2842 (94.5)
Within 7 days after delivery	566 (9.1)	20 (1.2)	366 (18.0)	36 (8.9)	166 (5.5)
**Prognosis status (%)**					
Live birth	487 (7.8)	11 (0.7)	335 (16.5)	22 (5.5)	140 (4.7)
Stillbirth	790 (12.7)	213 (13.3)	226 (11.1)	63 (15.6)	399 (13.3)
Dead within 28 days	189 (3.1)	13 (0.8)	79 (3.9)	11 (2.7)	97 (3.2)
Inducing labor	4751 (76.4)	1363 (85.2)	1389 (68.5)	308 (76.2)	2372 (78.9)
**Mother**					
**No. of pregnancy (%)**					
0	26 (0.4)	4 (0.3)	12 (0.6)	4 (1.0)	9 (0.3)
1	3221 (51.8)	818 (51.1)	1091 (53.8)	205 (50.7)	1560 (51.9)
≥2	2970 (47.8)	778 (48.6)	926 (45.6)	195 (48.3)	1439 (47.8)
**No. of live births (%)**					
0	2159 (34.7)	789 (49.3)	715 (35.2)	171 (42.3)	838 (27.9)
1	3165 (50.9)	641 (40.1)	1007 (49.6)	183 (45.3)	1692 (56.3)
≥2	893 (14.4)	170 (10.6)	307 (15.1)	50 (12.4)	478 (15.8)
**Age, years (%)**					
<20	184 (3.0)	54 (3.4)	75 (3.7)	13 (3.2)	83 (2.8)
20-25	1887 (30.4)	518 (32.4)	673 (33.2)	100 (24.8)	921 (30.6)
25-30	2141 (34.4)	517 (32.3)	667 (32.9)	155 (38.4)	1034 (34.4)
30-35	1190 (19.1)	317 (19.8)	349 (17.1)	88 (21.8)	562 (18.7)
≥35	815 (13.1)	194 (12.1)	265 (13.1)	48 (11.8)	408 (13.6)
**Race (%)**					
Han	5420 (87.2)	1393 (87.1)	1783 (87.9)	356 (88.1)	2608 (86.7)
Others	797 (12.8)	207 (12.9)	246 (12.1)	48 (11.9)	400 (13.3)
**Income level † (%)**					
<600 yuan	513 (8.3)	123 (7.7)	210 (10.4)	30 (7.4)	236 (7.9)
600-1200 yuan	980 (15.7)	269 (16.8)	356 (17.6)	59 (14.6)	457 (15.1)
1200-2400 yuan	1762 (28.3)	463 (28.9)	592 (29.2)	116 (28.7)	848 (28.2)
≥2400 yuan	2962 (47.6)	745 (46.6)	871 (42.8)	199 (49.3)	1467 (48.8)
**Education level (%)**					
Elementary school or less	511 (8.2)	144 (9.0)	191 (9.4)	29 (7.2)	223 (7.4)
Middle school	3916 (63.0)	1033 (64.6)	1337 (65.9)	242 (59.9)	1882 (62.6)
High school	996 (16.0)	218 (13.6)	309 (15.2)	64 (15.8)	527 (17.5)
College or above	794 (12.8)	205 (12.8)	192 (9.5)	69 (17.1)	376 (12.5)
**Infant**					
**Gestational age, week (%)**					
<28	3762 (60.5)	1436 (89.8)	1182 (58.3)	315 (78.0)	1416 (47.1)
28-37	1529 (24.6)	123 (7.7)	399 (19.7)	45 (11.1)	1132 (37.6)
≥37	926 (14.9)	41 (2.5)	448 (22.0)	44 (10.9)	460 (15.3)
**Birth weight, grams (%)**					
<2500	5028 (80.9)	1556 (97.3)	1514 (74.6)	356 (88.1)	2330 (77.5)
2500-4000	1119 (18.0)	43 (2.6)	483 (23.8)	46 (11.4)	639 (21.2)
≥4000	70 (1.1)	1 (0.1)	32 (1.6)	2 (0.5)	39 (1.3)
**Sex (%)**					
Male	2968 (47.7)	677 (42.3)	912 (45.0)	181 (44.8)	1537 (51.1)
Female	2985 (48.0)	768 (48.0)	1059 (52.1)	194 (48.0)	1414 (47.0)
Unknown	264 (4.3)	155 (9.7)	58 (2.9)	29 (7.2)	57 (1.9)
**Multiple births (%)**					
Yes	167 (2.7)	52 (3.3)	36 (1.8)	396 (98.0)	83 (2.8)
No	6050 (97.3)	1548 (96.7)	1993 (98.2)	8 (2.0)	2925 (97.2)

* Chi-square test was used to compare the characteristics of the four subtypes of neural tube defects (encephalocele was treated as reference group). Significant results of chi-square test are presented in red.

† Income level was presented as per person per year.

When the characteristics of the mothers and infants were examined by whether the NTD or subtype was accompanied by additional major defects (isolated versus non-isolated), significant differences were found for selected characteristics (Tables 4 and 5). For example, except for time to diagnosis, number of pregnancies, and race, significant differences were observed in all selected characteristics for NTDs. Additionally, non-isolated spina bifida was significantly easier to diagnose than isolated spina bifida during pregnancy. Similarly, live birth was more likely to be associated with isolated than non-isolated spina bifida. Notably, there was a significant difference in gestational age between NTDs and all the subtypes.

**Table 4:** Characteristics for neural tube defects, anencephaly, and spina bifida of infants and their mother (isolated *versus* non-isolated)

Characteristics	NTDs			Anencephaly			Spina bifida		
	Isolated	Non-isolated	*P* value	Isolated	Non-isolated	*P* value	Isolated	Non-isolated	*P* value
**No. of cases**	5356	861		1280	320		1035	994	
**Time of diagnosis (%)**			**0.76**			**0.26**			**<0.001**
During pregnancy	4866 (90.9)	785 (91.2)		1266 (98.9)	314 (98.1)		723 (69.9)	940 (94.5)	
Within 7 days after delivery	490 (9.1)	76 (8.8)		14 (1.1)	6 (1.9)		312 (30.1)	54 (5.5)	
**Prognosis status (%)**			**0.01**			**0.68**			**<0.001**
Live birth	433 (8.1)	54 (6.3)		10 (0.8)	1 (0.3)		292 (28.2)	43 (4.3)	
Stillbirth	675 (12.6)	115 (13.4)		166 (13.0)	47 (14.7)		94 (9.1)	132 (13.3)	
Dead within 28 days	150 (2.8)	39 (4.5)		10 (0.8)	3 (0.9)		54 (5.2)	25 (2.5)	
Inducing labor	4098 (76.5)	653 (75.8)		1094 (85.4)	269 (84.1)		595 (57.5)	794 (79.9)	
**Mother**									
**No. of pregnancy (%)**			**0.938**			**0.34**			**0.54**
0	23 (0.4)	3 (0.4)		4 (0.3)	0 (0)		8 (0.8)	4 (0.4)	
1	2776 (51.8)	445 (51.7)		649 (50.7)	169 (52.8)		553 (53.4)	538 (54.1)	
≥2	2557 (47.8)	413 (47.9)		627 (49.0)	151 (47.2)		474 (45.8)	452 (45.5)	
**No. of live births (%)**			**<0.001**			**0.30**			**<0.001**
0	1802 (33.6)	357 (41.5)		632 (49.4)	157 (49.1)		291 (28.1)	424 (42.7)	
1	2768 (51.7)	397 (46.1)		505 (39.5)	136 (42.5)		569 (55.0)	438 (44.1)	
≥2	786 (14.7)	107 (12.4)		143 (11.1)	27 (8.4)		175 (16.9)	132 (13.2)	
**Age, years (%)**			**<0.001**			**0.26**			**0.005**
<20	164 (3.1)	20 (2.3)		38 (3.0)	16 (5.0)		31 (3.0)	44 (4.4)	
20-25	1677 (31.3)	210 (24.4)		408 (31.9)	110 (34.4)		314 (30.3)	359 (36.1)	
25-30	1825 (34.1)	316 (36.7)		423 (33.0)	94 (29.4)		373 (36.0)	294 (39.6)	
30-35	1013 (18.9)	177 (20.6)		258 (20.1)	59 (18.4)		184 (17.8)	165 (16.6)	
≥35	677 (12.6)	138 (16.0)		153 (12.0)	41 (12.8)		133 (12.9)	132 (13.3)	
**Race (%)**			**0.61**			**0.91**			**0.54**
Han	4674 (87.3)	746 (86.6)		1115 (87.1)	278 (86.9)		914 (88.3)	869 (87.4)	
Others	682 (12.7)	115 (13.4)		165 (12.9)	42 (13.1)		121 (11.7)	125 (12.6)	
**Income level ^†^ (%)**			**<0.001**			**0.43**			**0.88**
< 600 yuan	467 (8.7)	46 (5.3)		93 (7.3)	30 (9.4)		112 (10.8)	98 (9.9)	
600-1200 yuan	868 (16.3)	112 (13.0)		221 (17.3)	48 (15.0)		184 (17.8)	172 (17.3)	
1200-2400 yuan	1555 (29.0)	207 (24.0)		375 (29.3)	88 (27.5)		300 (29.0)	292 (29.4)	
≥ 2400 yuan	2466 (46.0)	496 (57.7)		591 (46.1)	154 (48.1)		439 (42.4)	432 (43.4)	
**Education level (%)**			**<0.001**			**0.44**			**0.58**
Elementary school or less	447 (8.4)	64 (7.4)		119 (9.3)	25 (7.8)		103 (10.0)	88 (8.9)	
Middle school	3476 (64.9)	440 (51.1)		826 (64.5)	207 (64.7)		681 (65.8)	656 (66.0)	
High school	821 (15.3)	175 (20.3)		167 (13.1)	51 (15.9)		149 (14.4)	160 (16.1)	
College or above	612 (11.4)	182 (21.2)		168 (13.1)	37 (11.6)		102 (9.8)	90 (9.0)	
**Infant**									
**Gestational age, week (%)**			**<0.001**			**0.009**			**<0.001**
<28	3201 (59.8)	561 (65.2)		1156 (90.3)	280 (87.5)		487 (47.1)	695 (69.9)	
28-37	1338 (25.0)	191 (22.2)		87 (6.8)	36 (11.3)		202 (19.5)	197 (19.8)	
≥37	817 (15.2)	109 (12.6)		37 (2.9)	4 (1.2)		346 (33.4)	102 (10.3)	
**Birth weight, gram (%)**			**0.02**			**0.78**			**<0.001**
<2500	4309 (80.5)	719 (83.5)		1244 (97.2)	312 (97.5)		656 (63.4)	858 (86.3)	
2500-4000	980 (18.3)	139 (16.1)		35 (2.7)	8 (2.5)		353 (34.1)	130 (13.1)	
≥4000	67 (1.2)	3 (0.4)		1 (0.1)	0 (0)		26 (2.5)	6 (0.6)	
**Sex (%)**			**<0.001**			**0.17**			**<0.001**
Male	2541 (47.5)	427 (49.6)		556 (43.4)	121 (37.8)		482 (46.6)	430 (43.3)	
Female	2610 (48.7)	375 (43.6)		600 (46.9)	168 (52.5)		543 (52.5)	516 (51.9)	
Unknown	205 (3.8)	59 (6.8)		124 (9.7)	31 (9.7)		10 (0.9)	48 (4.8)	
**Multiple births (%)**			**<0.001**			**0.05**			**0.65**
Yes	128 (2.4)	39 (4.5)		36 (2.8)	16 (5.0)		17 (1.6)	19 (1.9)	
No	5228 (97.6)	822 (95.5)		1244 (97.2)	304 (95.0)		1018 (98.4)	975 (98.1)	

† Income level was presented as per person per year.

**Table 5:** Characteristics for encephalocele and congenital hydrocephalus of infants and their mother (isolated *versus* non-isolated)

Characteristics	Encephalocele			Congenital hydrocephalus		
	Isolated	Non-isolated	*P* value	Isolated	Non-isolated	*P* value
**No. of cases**	301	103		2039	969	
**Time of diagnosis (%)**			**0.09**			**0.94**
During pregnancy	270 (89.7)	98 (95.2)		1926 (94.5)	916 (94.5)	
Within 7 days after delivery	31 (10.3)	5 (4.8)		113 (5.5)	53 (5.5)	
**Prognosis status (%)**			**0.08**			**0.90**
Live birth	20 (6.6)	2 (1.9)		91 (4.5)	49 (5.1)	
Stillbirth	50 (16.6)	13 (12.6)		270 (13.2)	129 (13.3)	
Dead within 28 days	10 (3.3)	1 (1.0)		65 (3.2)	32 (3.3)	
Inducing labor	221 (73.5)	87 (84.5)		1613 (79.1)	759 (78.3)	
**Mother**						
**No. of pregnancy (%)**			**0.02**			**0.27**
0	4 (1.3)	0 (0)		4 (0.2)	5 (0.5)	
1	142 (47.2)	63 (61.2)		1051 (51.5)	509 (52.5)	
≥2	155 (51.5)	40 (38.8)		984 (48.3)	455 (47.0)	
**No. of live births (%)**			**0.03**			**<0.001**
0	117 (38.9)	54 (52.4)		464 (22.8)	374 (38.6)	
1	142 (47.1)	41 (39.8)		1246 (61.1)	446 (46.0)	
≥2	42 (14.0)	8 (7.8)		329 (16.1)	149 (15.4)	
**Age, years (%)**			**0.18**			**0.09**
<20	10 (3.3)	3 (2.9)		51 (2.5)	32 (3.3)	
20-25	75 (24.9)	25 (24.3)		603 (29.5)	318 (32.8)	
25-30	106 (35.2)	49 (47.6)		725 (35.6)	309 (31.9)	
30-35	70 (23.3)	18 (17.5)		392 (19.2)	170 (17.5)	
≥35	40 (13.3)	8 (7.7)		268 (13.2)	140 (14.5)	
**Race (%)**			**0.43**			**0.48**
Han	263 (87.4)	93 (90.3)		1774 (87.0)	834 (86.1)	
Others	38 (12.6)	10 (9.7)		265 (13.0)	135 (13.9)	
**Income level ^†^ (%)**			**0.80**			**0.006**
< 600 yuan	22 (7.3)	8 (7.8)		164 (8.0)	72 (7.4)	
600-1200 yuan	47 (15.6)	12 (11.7)		280 (13.7)	177 (18.3)	
1200-2400 yuan	86 (28.6)	30 (29.1)		569 (27.9)	279 (28.8)	
≥ 2400 yuan	146 (48.5)	53 (51.4)		1026 (50.4)	441 (45.5)	
**Education level (%)**			**0.41**			**0.21**
Elementary school or less	24 (8.0)	5 (4.9)		137 (6.7)	86 (8.9)	
Middle school	184 (61.1)	58 (56.3)		1288 (63.2)	594 (61.3)	
High school	44 (14.6)	20 (19.4)		357 (17.5)	170 (17.5)	
College or above	49 (16.3)	20 (19.4)		257 (12.6)	119 (12.3)	
**Infant**						
**Gestational age, week (%)**			**0.03**			**<0.001**
<28	228 (75.8)	87 (84.5)		840 (41.2)	576 (59.4)	
28-37	33 (11.0)	12 (11.7)		867 (42.5)	265 (27.4)	
≥37	40 (13.2)	4 (3.8)		332 (16.3)	128 (13.2)	
**Birth weight, gram (%)**			**0.014**			**<0.001**
<2500	257 (85.4)	99 (96.1)		1537 (75.4)	793 (81.8)	
2500-4000	42 (14.0)	4 (3.9)		468 (23.0)	171 (17.7)	
≥4000	2 (0.6)	0 (0)		34 (1.6)	5 (0.5)	
**Sex (%)**			**0.23**			**<0.001**
Male	128 (42.5)	53 (51.5)		1088 (53.4)	449 (46.3)	
Female	152 (50.5)	42 (40.8)		922 (45.2)	492 (50.8)	
Unknown	21 (7.0)	8 (7.7)		29 (1.4)	28 (2.9)	
**Multiple births (%)**			**0.14**			**0.37**
Yes	4 (1.3)	4 (3.9)		60 (2.9)	23 (2.4)	
No	297 (98.7)	99 (96.1)		1979 (97.1)	946 (97.6)	

† Income level was presented as per person per year.

xxx

## DISCUSSION

This population-based study is one of the few reports from China not only to describe the time trends in the prevalence of NTDs and their subtypes during the past decade but also to identify the epidemiological characteristics associated with these malformations. From 2006 to 2015, the prevalence of NTDs in Liaoning Province in northeast China significantly decreased from 28.8 per 10,000 live births to 7.5 per 10,000 live births. A similar pattern was also observed for all the subtypes. When stratified by whether the malformation was accompanied by additional major defects (isolated versus non-isolated), we found significant differences in selected characteristics (e.g., prognosis status, gestational age, and birth weight). Compared with those in several developed countries, higher prevalence rates were still detected in this population, especially for congenital hydrocephalus and spina bifida. This result indicates that further prevention efforts are warranted to reduce the future risk of NTDs.

Our study found that the overall prevalence of NTDs in Liaoning Province from 2006 to 2015 was 19.1 per 10,000 live births [7]. When compared with Europe, the prevalence found in the present study was intermediate between the higher rate reported in Turkey (35.9 per 10,000 live births) by Onrat and colleagues [11] in 2009 and the lower rate reported in Spain (1.3 per 10,000 live births) using the data in the EUROCAT network [12] for 2012. Furthermore, the prevalence for other provinces in China ranged from 0.3 per 10,000 live births reported in Beijing by Li and colleagues [9] in 2009 to 199.4 per 10,000 live births reported in Shanxi Province by Chen and colleagues [8] in 2009. Regarding the time trend in NTDs prevalence in Liaoning province, except for encephalocele, the prevalence of the other NTDs subtypes fluctuated but had an obvious downward trend after 2009, which was in accordance with the results of a study conducted by Liu and colleagues [10]. Additionally, the significantly decreasing trend in prevalence for isolated rather than non-isolated NTDs found in the present study was consistent with that observed in a previous study conducted by Collins and colleagues [13].

A previous study carried out by Li and colleagues [1] showed that based on data gathered from urban and rural areas in the south of China the total prevalence of NTDs from 2006 to 2008 was 9.7 per 10,000 births. We found that the prevalence of NTDs in Liaoning Province (northern area of China) was relatively higher during the same period (27.16 per 10,000 live births). Although many studies have attempted to explain geographical differences, the exact reasons for such discrepancies are unclear. Previous studies have demonstrated that the mutation frequency of methylene tetrahydrofolate reductase C677T among people in the north of China was higher than that in the south, and there is a higher risk of NTDs among fetuses with this mutated gene [14, 15]. Nevertheless, a single genetic mutation is inadequate to explain the aforementioned difference. Additionally, several risk factors, including air pollution and diet, may partly contribute to this difference. For example, the main fuel used for energy processes in the north is coal, which can generate more indoor air pollution than there is in the south [16]. Li and colleagues [17] demonstrated that the risk of NTDs increases with increasing exposure indices, showing a dose-response trend in a case-control study. As for outdoor air pollution, Padula and colleagues [18] showed that ambient air pollution and traffic exposure in early gestation contributed to the risk of NTDs from 1997 to 2006. Furthermore, fewer green vegetables and more pickled vegetables are consumed in the north, and this may result in folic acid and vitamin deficiency, which could be important reasons for a higher prevalence of NTDs in northern than in southern China [1]. These factors may contribute to different NTD prevalence rates among different regions, but additional studies should be conducted to definitively determine the causes.

Although termination of pregnancy for fetal anomaly has considerably reduced the live birth prevalence of these malformations, it is not an optimal solution for NTDs that are preventable with readily available and low cost interventions, as is the case for NTDs with folic acid supplementation or food fortification [19]. Canada and the United States first launched mandatory fortification of 140 μg of folic acid per 100 g of enriched cereal grain products. After this policy was enacted, many studies documented a reduction in malformations [20]. A nationwide folic acid supplementation program was initiated in China in 2009. This program provides free folic acid supplements to all women who have a rural registration and plan to become pregnant [21]. We found that the prevalence of NTDs from 2012 to 2015 decreased markedly from that of 2009, which may be due in part to the effects of this national policy. Although not all subtypes showed a statistically significant decreasing trend in prevalence rates, for example, the prevalence of encephalocele, including isolated and non-isolated cases, was not significantly decreased, this phenomenon might be merely attributable to the limited number of cases examined here. Therefore, policies for mandatory fortification with folic acid should be considered as an important and effective pathway for prevention of NTDs, and supplementation with adequate folic acid for pregnant women should be recommended to decrease the prevalence of NTDs.

In our study, we classified NTDs as non-isolated or isolated depending on whether or not they were accompanied by additional major defects, respectively. We found that the majority of NTDs cases in Liaoning Province were isolated (81.3%). Khoury and colleagues [22] found that isolated NTDs were more sensitive to environmental factors, whereas non-isolated NTDs were sensitive to both environmental and genetic factors. Other research has also revealed a significant decline in the United States between 1992 and 2009, since supplementation with folic acid in 1998, for the prevalence of isolated but not non-isolated NTDs [13]. Therefore, the evidence suggests that folic acid deficiency may affect isolated NTDs to a great extent [8]. Thus, our study supports the idea that it is important to distinguish isolated and non-isolated NTDs due to different epidemiological characteristics. However, more attention should be focused on non-isolated NTDs because of the complicated pathogenic processes and severe clinical manifestations. The prevalence of non-isolated cases was barely decreased simply by intake of folic acid; hence, additional prevention efforts should be undertaken to reduce non-isolated NTDs.

Our study has several strengths. Previous to the present study, only a few studies have reported the prevalence of NTDs in China. We conducted a large-scale study focusing on long-term trends of NTDs to comprehensively describe the time trends in the prevalence of NTDs based on data from a number of population-based registries in Liaoning Province, one of the most important provinces in China. We further evaluated the trends in the prevalence for all major NTDs subtypes and identified the epidemiological characteristics, including whether the NTD was isolated or non-isolated, to provide detailed information about these malformations.

Despite the clear strengths of our study, some limitations should be acknowledged. First, we had no access to the demographic factors for all live births in Liaoning Province, which hindered our ability to investigate the potential causes of the trends. Second, the policy of mandatory premarital physical check-ups became voluntary throughout the country on October 1, 2003 [10]. However, we were unable to access NTD prevalence data in Liaoning Province prior to 2006. Therefore, we could not evaluate whether this policy change affected the change in prevalence for NTDs. Third, we could not exclude the possibility that registration problems might exist for the data used in our study. The NTDs were diagnosed in different hospitals and cities in Liaoning Province, which might have generated bias in our study. Although there have already been some quality control measures, potential “under-reporting” and “misclassification” of the birth defects are difficult to be avoided and may still occur in the registry system, especially in those less developed areas with poor medical conditions. Lastly, the maximum time to diagnosis for NTDs was the seventh day after birth. We did not include NTDs confirmed after the seventh day, which might have led to slightly lower prevalence rates in our study than in studies that include longer periods for confirmed diagnoses.

In conclusion, this population-based study provided the most recent and detailed evidence for time trends in the prevalence of NTDs, as well as epidemiological characteristics, in one of the largest provinces of China from 2006 to 2015. The decreasing trend we found in NTD prevalence may be partly attributed to the nationwide folic acid supplement program as a public health strategy to prevent NTDs. However, the prevalence of NTDs in Liaoning Province remains higher than that in the south of China or several developed countries, which draws attention to the necessity of improving the efficiency of the periconceptional folic acid supplementation program during pregnancy. Future prevention efforts should be strengthened to reduce the risk of NTDs, especially for the non-isolated subtype, in areas with continued high prevalence.

## MATERIALS AND METHODS

### Study population and data source

Liaoning Women and Children’s Health Hospital is one of the few obstetrical and gynecological hospitals for the province of Liaoning. It has also been a comprehensive care institution, responsible for women and children’s health-care guidance. Data from 2006 to 2015 were retrieved from the maternal and child health certificate registry of Liaoning Province, which was maintained by this hospital. Hospital-delivered live-born and stillborn infants are included in this registry. This registry covers all 14 cities of the province (Shenyang, Dalian, Anshan, Fushun, Benxi, Dandong, Jinzhou, Yingkou, Fuxin, Liaoyang, Panjing, Tieling, Chaoyang, and Huludao), with approximately 42 million inhabitants. Liaoning Province is one of the 31 provinces providing data to the national birth defects surveillance database maintained by the Chinese Birth Defects Monitoring Network. All congenital malformation data are regularly uploaded to the online reporting system for maternal and child health surveillance by specialized staff in Liaoning Women and Children’s Health Hospital [23, 24]. The maximum time to provide a diagnosis of a congenital malformation is the seventh day after birth [25].

### Data collection

Detailed procedures for data collection have been described in a previous report [26, 27]. Briefly, provincial and city surveillance networks as well as clinical expert groups were established to undertake the data collection. Each neonate (or terminated fetus) was examined immediately after birth by trained health-care professionals, to screen for congenital malformations. For suspected cases that were diagnosed through prenatal ultrasound scans, case ascertainment after termination or examination after the birth was requested. Once an NTD case was identified and confirmed at the monitored hospital by experts in the department of pediatrics or obstetrics or through an ultrasound, the mother of the infant was interviewed by the staff to complete the “Birth Defects Registration Form.” This form was used for collecting information, including demographic characteristics, clinical features, and obstetric factors. Subsequently, the form was submitted first to the local maternal and child health facility and then to the provincial maternal and child health hospital, which was Liaoning Women and Children’s Health Hospital. Data from these cases were reviewed and confirmed by a group of state-level experts in medical genetics and pediatrics [25].

### NTD classification

According to the World Health Organization’s diagnostic tool the International Classification of Diseases, 10th Revision (ICD-10), NTDs include four major congenital malformations: anencephaly (ICD10: Q00), spina bifida (ICD10: Q05), encephalocele (ICD10: Q01), and congenital hydrocephalus (ICD10: Q03). We further classified NTDs into isolated and non-isolated NTDs, with the latter defined as NTDs with additional major defects. Isolated NTDs were divided into single NTDs with malformations at one site, and multiple NTDs with malformations at two or more sites [8]. Single NTDs included anencephaly, spina bifida, encephalocele, and congenital hydrocephalus. The birth prevalence of NTDs was expressed as the number per 10,000 live births. The denominator was based exclusively on the total number of live births, using data obtained primarily from the Liaoning Women and Children’s Health Hospital. The total number of live births in the study window was 3,248,954, with 6217 cases of NTD identified.

Quality control of the data has been described previously in detail [25]. Briefly, according to the program manual to ensure high quality data, the disease diagnosis, data collection, data checking, and medical records were verified by the expert group at each level. In addition, an independent retrospective survey was organized by the experts to find deficiencies and inaccuracies in the data [25].

### Statistical analysis

NTD prevalence rates were calculated for nine 1-year time intervals from 2006 to 2015. In order to specifically examine time trends, the Poisson regression model was used to find the line of best fit for NTD prevalence by year, with year entered into the model as a continuous independent variable. Percentages were calculated for selected variables of NTDs with or without other major malformations. Categorical variables were compared using Pearson χ^2^ tests. All analyses were conducted using SPSS software for Windows (version 23, SPSS Inc., Chicago, IL, USA). All statistical tests were two-sided, and *P*-values less than 0.05 were considered statistically significant.

Qi-Jun Wu was supported by the Fogarty International Clinical Research Scholars and Fellows Support Center at the Vanderbilt Institute for Global Health, funded by the Fogarty International Center, NIH, through an R24 Training Grant (D43 TW008313 to Xiao-Ou Shu).

T-NZ and T-TG contributed equally to this work.
